# HSSNet: A End-to-End Network for Detecting Tiny Targets of Apple Leaf Diseases in Complex Backgrounds

**DOI:** 10.3390/plants12152806

**Published:** 2023-07-28

**Authors:** Xing Gao, Zhiwen Tang, Yubao Deng, Shipeng Hu, Hongmin Zhao, Guoxiong Zhou

**Affiliations:** College of Computer & Information Engineering, Central South University of Forestry and Technology, Changsha 410004, China; 20202756@csuft.edu.cn (X.G.); 20202898@csuft.edu.cn (Z.T.); 20202882@csuft.edu.cn (Y.D.); 20211100456@csuft.edu.cn (S.H.)

**Keywords:** apple leaf diseases, complex background, tiny-object detection, HSSNet, TTALDD-4

## Abstract

Apple leaf diseases are one of the most important factors that reduce apple quality and yield. The object detection technology based on deep learning can detect diseases in a timely manner and help automate disease control, thereby reducing economic losses. In the natural environment, tiny apple leaf disease targets (a resolution is less than 32 × 32 pixel^2^) are easily overlooked. To address the problems of complex background interference, difficult detection of tiny targets and biased detection of prediction boxes that exist in standard detectors, in this paper, we constructed a tiny target dataset TTALDD-4 containing four types of diseases, which include Alternaria leaf spot, Frogeye leaf spot, Grey spot and Rust, and proposed the HSSNet detector based on the YOLOv7-tiny benchmark for professional detection of apple leaf disease tiny targets. Firstly, the H-SimAM attention mechanism is proposed to focus on the foreground lesions in the complex background of the image. Secondly, SP-BiFormer Block is proposed to enhance the ability of the model to perceive tiny targets of leaf diseases. Finally, we use the SIOU loss to improve the case of prediction box bias. The experimental results show that HSSNet achieves 85.04% mAP (mean average precision), 67.53% AR (average recall), and 83 FPS (frames per second). Compared with other standard detectors, HSSNet maintains high real-time detection speed with higher detection accuracy. This provides a reference for the automated control of apple leaf diseases.

## 1. Introduction

Apples have been widely grown worldwide because of their ecological adaptability, high nutritional content and economic profit. Influenced by natural environmental factors, apple leaves are susceptible to infection by a variety of diseases. These diseases seriously impede the normal metabolic process of apples (e.g., leaf photosynthesis, respiration, and transpiration), causing leaf decay and even leaf loss, which leads to a decrease in fruit quality and yield [1,2]. Therefore, the phenotypic characteristics exhibited by leaves during apple growth and development have important applications for screening diseases, inhibiting their spread, and reducing economic losses. The traditional manual apple leaf disease detection methods have the disadvantages of low efficiency and high workload, and they require the professionalism of the inspectors [3]. There is an urgent need for a stable automated apple leaf disease detection method to replace manual work. In recent years, with the rapid development of computer hardware and machine vision technology, the application of object detection technology to apple leaf disease detection has become the development trend of modern apple cultivation [4,5].

Currently, in apple leaf disease detection research, traditional machine vision methods based on machine learning and pattern recognition have achieved certain results under restricted situations. For example, certain detection and recognition results can be achieved in a laboratory environment (uniform illumination and simple background environment) [6,7,8]. However, such a standard environment controls the interference of natural background, and the trained detectors are vulnerable to external noise interference in real-application conditions. In addition, there are few types of diseases that can be identified by these methods, which is difficult to put into the natural environment applications.

Recently, deep learning-based methods have begun to be widely discussed and studied in crop disease identification [9,10]. Deep learning-based object detection algorithms do not need to extract image features by manually constructing operators, and the extracted features are more robust. In the early days of deep learning-based object detection research [11], deep learning-based object detection algorithms commonly adopted the Two-Stage approach, which explicitly divides the detection process into two stages: region proposal selection and target region selection. These methods are represented by R-CNN (region-based convolutional neural network) [12,13] and its variants, which can obtain high detection precision, but whose detection speed is slow. In 2016, the One-Stage object detection method, represented by YOLOv1 [14,15], gained widespread interest and gradually became a mainstream universal detector due to its strong feature-extraction capability, high scalability, and fast detection speed. Instead of outputting candidate regions in advance, the One-Stage method outputs the class probability and location of the object in an end-to-end fashion. This greatly optimizes the overhead of computational resources and improves the efficiency of detection. So far, the YOLO algorithm family has been evolved to version v8 [16,17]. It is worth mentioning that the YOLO family of benchmark networks still suffers from weak global information modeling capability and poor detection of tiny targets.

Following an existing survey [3,18,19], YOLO detectors are widely used for crop pest detection in uncontrolled environments (e.g., tea plantations and orchards). YOLO series detectors can achieve high detection accuracy while ensuring the need for real-time detection. In 2021, Mathew et al. [20] built a UAV platform to capture video and images of fruit trees on farms and subsequently built their own apple leaf disease dataset. They successfully detected three common diseases, Apple Scab, Cedar Apple Rust, and Black rot, with high precision using the original YOLOv3 algorithm. However, the appearance characteristics of the diseases were not considered. They were not tailored to design apple leaf, disease-specific network models. In 2022, the YOLOX-ASSANano [21] lightweight model based on YOLOX-Nano was designed for real-time apple disease detection. The model showed a performance of 58.85% mAP at 122 FPS on the public dataset PlantDoc. In 2022, Li et al. [22] proposed Apple-YOLO, a mobile-device-oriented apple leaf disease detection model based on YOLOv5. The model can automatically extract features of different disease spots and conduct early phenotypic characterization of eight different diseases for rapid detection.

The aforementioned studies achieved good results and demonstrated the viability of the YOLO family of algorithms for leaf disease detection tasks. However, these studies mainly focused on issues such as improving model inference speed, reducing the number of model parameters, and not using the state-of-the-art YOLO detector. The influence of tiny target characteristics of the disease and the complex background of natural environments on detection models needs to be thoroughly explored. For this reason, we chose the latest YOLOv7 as the benchmark network to carry out related research. To this end, we chose the latest YOLOv7-tiny as a benchmark network to carry out related work to explore the possibility of deep learning to optimize apple leaf disease detection. This paper focuses on four types of apple leaf diseases, Alternaria leaf spot, Frogeye leaf spot, Grey spot and Rust disease. Their detailed properties and negative effects will be presented in Section 2.1.

**Figure 1:**
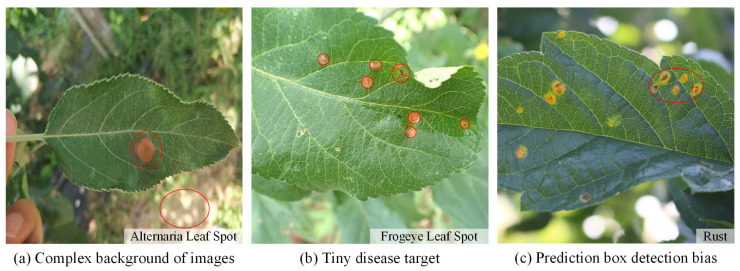
Problems with apple leaf disease detection.

In the current apple leaf disease detection process (e.g., Figure 1), there are three problems that urgently need to be addressed:

(a) Complex image background. Apple leaf images in real environments have complex background environments, natural illumination conditions, or shadow occlusion. The chromaticity and appearance of the background environment may be similar to the foreground features of the disease. This can interfere with the feature parameters of the network, interfere with the supervised learning process, and pose a great challenge to the detection network.

(b) Tiny disease targets. The spot texture of tiny target diseases occupies a small area on the leaf and is sparsely distributed. The feature information domain is limited and difficult for the network to capture accurately. The existing standard detectors do not guide the model to focus on these features and are prone to missed detection.

(c) Prediction box detection bias. When some tiny target diseases are clustered, the YOLO object detection algorithm tends to generate more dense redundant frames in the prediction process, and the loss function does not consider the mismatch direction between the real frame and the predicted frame. This can lead to difficulty in quickly calibrating the exact location of tiny target diseases, slowing down the training speed and impairing the detection precision.

For the problem of complex background, the traditional concept uses filters with hand-constructed operators to reduce background noise [23,24] in order to highlight the foreground target features. Another kind of method is to extract the significant regions of lesions in the complex background by preprocessing [25]. However, most of the above methods are multi-stage, which is not conducive to automating the processing process, and the algorithms are simple and constrained, making it difficult to be effectively promoted. For the whole image, the network should be guided to prioritize the primary leaf disease object information by giving them higher weights while weakening irrelevant signals. The attention module has been demonstrated to perform this task well [26]. The essence of the mechanism is to focus the part of interest by a set of weighting coefficients learned through the network structure and weighed in a way that suppresses weakly associated background regions. The existing attention modules, e.g., SE (squeeze-and-excitation) [27] and CBAM (convolutional block attention module) [28] are mostly based on neural networks of one-dimensional channel type or two-dimensional spatial type. Their architectures are built by a series of complex convolution and pooling operations, which require additional parameter overhead and lack interpretability. To address these issues, we propose H-SimAM (simple, parameter-free attention module) to enhance the ability of detection models to focus on apple leaf disease characteristics [29]. It is constructed based on three-dimensional weights (i.e., those considered across spatial and channel dimensions) and energy functions, and does not add additional network parameters.

In the existing studies, the most general definition of tiny targets comes from MS COCO, a common dataset in the field of object detection [30]. It defines a tiny target in terms of absolute scale with an image resolution of less than 32 × 32 pixel^2^. To address the problem of tiny object detection, some researches increase the network’s ability to fit tiny object features by training strategies such as oversampling and data augmentation [31]; some researches highlight the importance of small objects by reducing the number of large objects [32]; others increase the resolution of the feature map of small object regions to detect smaller objects. The emergence of the Transformer architecture provides new thoughts to solve the small object [33]. The Transformer architecture can encode the contextual information of tiny targets, fuse more correlation information, and enhance the model’s perception of tiny targets. Therefore, we propose the SP-BiFormer Block to optimize the ability of YOLOv7 to detect tiny target disease features. SP-BiFormer is implemented based on Biformer [34] with dynamic sparse perception capability and SoftPool [35] with a soft property of the downsampling process, which has the ability to extract apple leaf disease tiny target features with excellent performance.

To address the problem of more redundant boxes in the prediction process for tiny targets of disease, the classical IOU loss cannot measure the overlap between the predicted and real boxes well, so it is more biased towards objects of larger size in the training process. GIOU [36] introduced the minimum external matrix on top of IOU to express the intersection property of two boxes more accurately. DIOU [37] directly regressed the Euclidean distance between the centroids of the two boxes, which accelerated the model’s convergence. However, the aspect ratio of the bounding box is not considered in the regression process, and a further improvement in accuracy is still needed. None of the above loss functions measure the direction of the mismatch between the predicted box and the real box. For this reason, we use SIOU loss [38] as the loss function, which defines the angle cost and introduces it into the distance cost, thus prompting the regression process to efficiently perform the anchor box calibration of the tiny target of the disease.

To address the foregoing issues, in this paper, we propose a novel object detection network, HSSNet, focusing on the detection of tiny target apple leaf diseases, using YOLOv7-tiny as a benchmark.

(1) We construct an image dataset of the tiny target apple leaf diseases for detection (TTALDD-4), which contains Alternaria leaf spot, Frogeye leaf spot, Grey spot and Rust four categories of apple leaf diseases, and 6482 images in total. Among them, 5998 images contained complex backgrounds, accounting for 92.55% of all disease areas in TTALDD-4, were accurately labeled with location boxes and corresponding disease category labels. There was a total of 31,914 target boxes and 22,506 small target boxes, accounting for 70.52% of all disease areas.

(2) The main improvements of HSSNet are as follows:

(a) For the problem of complex apple leaf disease background, we propose the H-SimAM attention mechanism. It enhances the expression of neural signals that are highly correlated with disease targets, and suppresses interfering signals. H-SimAM improves the ability of YOLOv7-tiny to focus on foreground leaf lesions in complex backgrounds.

(b) For the problem of disease tiny object detection, we propose the SP-BiFormer Block. This block is based on the BRA module with dynamic sensing queries and SoftPool, which enhances the ability of YOLOv7-tiny to capture the sparse distribution of tiny targets of leaf diseases.

(c) To address the problem of biased prediction frame detection, we use the SIOU loss function to optimize the training process. This loss function takes into account the mismatch direction between the prediction frame and the real frame to make the training process fit better with the dense distribution characteristics of tiny targets of apple leaf diseases.

(3) The experimental results show that the detection accuracy of HSSNet is significantly improved compared with the baseline network YOLOv7-tiny. The proposed model achieves a good tradeoff between accuracy, detection speed, and parameters.

## 2. Materials and Methods

### 2.1. Data Acquisition and Processing

Suitable image datasets are required to train the object detection network. The AppleLeaf9 dataset [39] provides a useful reference for us as a multi-source fusion dataset including PVD, ATLDSD, PPCD2020, and PPCD2021 for healthy apples and eight apple leaf diseases. A total of 94% of this dataset contains images of the wild environment, which improves the robustness of the model during real-world applications. In addition, AppleLeaf9 uses the CLAHE [40] technique to reduce the noise in the images and improves the diversity of the images through data enhancement. Therefore, we constructed an image dataset of the tiny target apple leaf diseases for detection (TTALDD-4) based on the definition of COCO tiny targets in AppleLeaf9 by selecting a total of four types of apple small target diseases, namely, Alternaria leaf spot disease, Frogeye leaf spot disease, Grey spot disease, and Rust disease. The dataset contains a total of 6481 images, with 5998 complex backgrounds, accounting for 92.55%.

**Figure 2:**
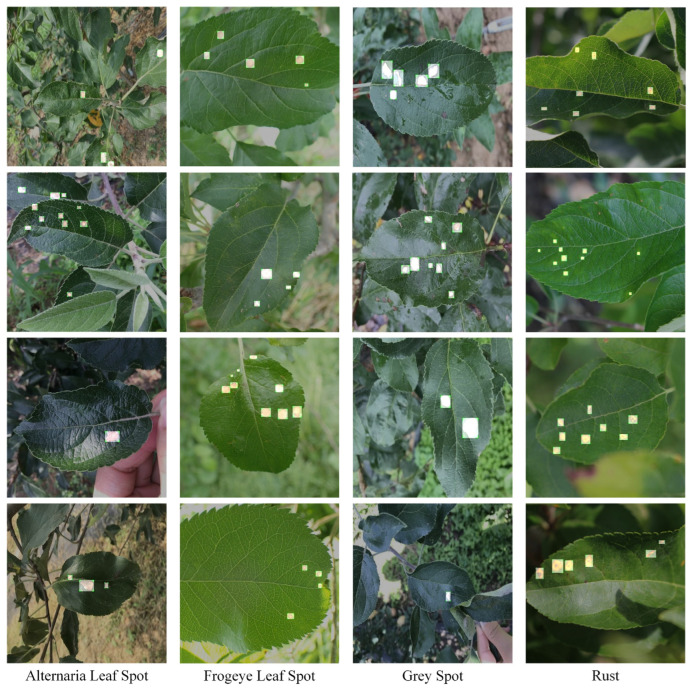
Schematic diagram of the location label.

Supervised learning for object detection training requires not only the category labels but also the localization labels of the diseases. Therefore, we used the Labelimg software (v1.8.1) to accurately label disease areas under the guidance of Yahui Hu [26] from the Hunan Academy of Agricultural Sciences. Specifically, when labelling an image, we first use a rectangular box to label the exact region of the disease and then add the category to which the disease belongs. The edges of this rectangular box fit the geometric edges of the disease to ensure the accuracy of supervised learning. After the labelling is complete, the information about the disease in that image is recorded via an XML file format, which contains the coordinate position of the rectangular box and the category of the disease to which it belongs. Finally, we obtained 31,914 target boxes, and the tiny target boxes (pixel area less than 32 × 32 pixel^2^) are 22,506, accounting for 70.52%. In addition, during the labeling process, we removed the very blurred images to facilitate the effective training of the network, and the number of images and labels obtained are statistically shown in Table 1. In the Image Num column of the table, the front number represents the number of images, and the back one represents the number of images with complex backgrounds; in the Label Num column, the former represents the total number of labels, and the latter represents the number of labels for small targets. Figure 2 shows the results of the labeled boxes.

In order to improve the expertise of the detector in detecting phenotypic characteristics of tiny target diseases of apple leaves, we analyzed the four categories of diseases mentioned above. Healthy apple leaves are overall ovate or oval in shape, with serrated leaf margins, well-defined vein textures, and are usually soft or dark green in color. Alternaria leaf spot disease of apples caused by Streptomyces infection is a devastating disease for apple production. Infected leaves show dark brown or black leaf spots, and these cells rapidly spread and accumulate into necrotic lesions that eventually cause leaf abscission [2]. Frogeye leaf spot disease lesions are generally tan in color, and these lesions are distributed throughout the leaf in dots or patches. Grey spot disease initially appears as subrounded lesions with well-defined margins that are red-brown. Later, it turns gray with small black spots scattered in the center and the spots are slightly sunken. It usually occurs at the same time as rust disease, with a peak incidence in autumn. Rust disease is a fungal disease that produces small, shiny spots with an orange-red color on the lesions [41]. Rust disease can spread to other soft green tissues of apple trees, such as shoots and new tips, in severe cases. It often causes the withering of apple leaves and reduced photosynthetic function.

All four types of diseases have tiny areas of incidence and lesion traits that are more easily confused with the leaf background. All these problems make the standard detector performance low. Failure to detect diseases in a timely manner will seriously affect apple production and make economic benefits significantly impaired. Therefore, the research of these representative apple leaf disease tiny targets has practical application value for apple leaf pest control.

### 2.2. The Structure of HSSNet

**Figure 3:**
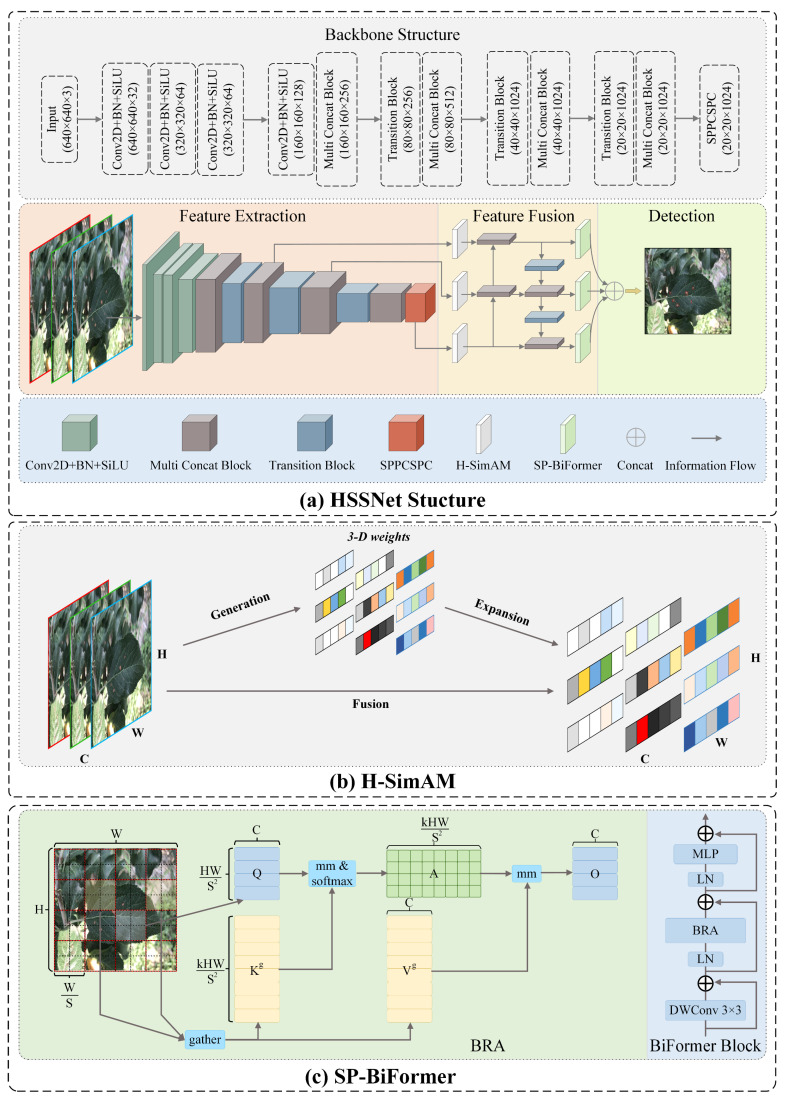
The structure of HSSNet.

In this paper, a more specialized detection model, HSSNet, is proposed for the characteristic of tiny target diseases of apple leaves. Its network architecture is shown in Figure 3a. Specifically, a 640 × 640 image of the disease is input and feature extraction is performed in the backbone network, and the associated dimensional changes are shown in Figure 3. Before the extracted multi-scale features enter the feature pyramid, we use the H-SimAM zero-parameter attention module to filter the noisy signals in the complex background. This mechanism is based on neuroscience theory and enhances the representation of signals with strong correlation to disease targets without introducing additional parameters to the network. In the pyramid, multi-scale features are fused through the upper and lower information paths. Before the fused features enter the detection head, we embed the SP-BiFormer module to enhance the ability of the network to focus on tiny targets of the disease. This module has sparse perception capabilities and associates the perception features of tiny target disease and context, which increases the amount of information of small target location and category features. In addition, we use SIOU loss to optimize the model training. SIOU takes into account the direction of mismatch between predicted and real frames, which makes the model training more suitable to the characteristics of the aggregated distribution of diseases and improves the detection performance. In the following, we describe the detailed techniques of the components of HSSNet.

#### 2.2.1. Hard Simple Parameter-Free Attention Module (H-SimAM)

In the natural environment, the precise location of apple leaf disease is difficult to capture directly by sensing devices. Areas of lesions under natural lighting are not well distinguished and can easily be confused with similar backgrounds, leading to missed detections. In addition, the color of certain diseases is not well differentiated from the planting background or the leaves themselves, and the features abstracted by convolutional networks are easily disturbed by background noise. These complex natural environments place high demands on the model to accurately focus on disease features. The attention module in machine vision can be understood as focusing on the part of the input that is expected to be attended to and suppressing the unimportant interference signals.

As mentioned in the introduction, the SE Attention Module is a classical attention mechanism for channel dimension. It first performs the global average pooling operation on the input feature map to compress the global feature volume, and then obtains the importance of each channel of the feature map through the excitation operation, so that the neural network can focus on certain feature channels. It consists of two submodules: the CAM (channel attention module) and the SAM (spatial attention module). The CAM module compresses the information in the spatial dimension and focuses more on the information in the input image that is meaningful to the task. SAM compresses the information in the channel dimension and the module focuses on the location information of the target. The above attention modules do not pay differential attention to different feature maps or neurons in spatial locations, which may limit their expressive power. In addition, these attention modules inevitably increase the parameters and computational complexity while improving network detection.

For this purpose, we propose a parameterization-free attention module, H-SimAM, as shown in Figure 3b. It is an improved version of SimAM, which is a product of neuroscience theory [42], and is more adaptable to the detection of apple leaf pests and diseases. It is worth emphasizing that this module is a small plug-and-play component with generality.

To select the more important feature signals, SimAM estimates the importance of individual neurons (computational units) by means of an energy function:

(1) $$e_{t}\left({t,x}_{i},w_{t},b_{t}\right)=\frac{1}{M-1}\sum_{i=1}^{M-1}{\left(-1-\left(w_{t}x_{i}+b_{t}\right)\right)}^{2}+{\left(1-\left(w_{t}t+b_{t}\right)\right)}^{2}+\varphi w_{t}^{2},$$

where, t and $x_{i}$ are the target and other computational units, respectively, in a single channel with input feature $X\in \;R^{C\times H\times W}$. i  is the index of other computational units in the spatial dimension and M is the number of computational units on a single channel. $w_{t}$ and $b_{t}$ are the weights and biases of the output of the computational units in the linear transformation. The values 1 and −1 are binary labels for simple settings. $\varphi w_{t}^{2}$ is the canonical term.

Through minimizing the above equations, the linear differentiability of t and other computational units in the same channel can be determined, thus enabling the evaluation of the importance of computational units. The parameters $w_{t}$ and $b_{t}$, can be determined by the following analytical solution:

(2) $$\left\{\begin{array}{c} w_{t}=-\frac{2\left(t-{µ}_{t}\right)}{{\left(t-{µ}_{t}\right)}^{2}+2\sigma_{t}^{2}+2\varphi }\\ b_{t}=-\frac{1}{2}\left(t+{µ}_{t}\right)w_{t} \end{array}\right.,$$

where ${µ}_{t}=\frac{1}{M-1}\sum_{i=1}^{M-1}x_{i}$ and ${2\sigma }_{t}^{2}=\frac{2}{M-1}\sum_{i=1}^{M-1}{{(x}_{i}-{µ}_{t})}^{2}$ are the mean and variance of the output of all computational units except t, respectively.

Assume that the values in the same channel have the same distribution pattern. Given this assumption, the minimum energy of the input features in both H and W dimensions can be calculated using the following equation [29]:

(3) $$e_{t}^{min}=\frac{4\left({\hat{\sigma }}^{2}+\varphi \right)}{{\left(t-\hat{µ}\right)}^{2}+2{\hat{\sigma }}^{2}+2\varphi },$$

where $\hat{µ}=\frac{1}{M}\sum_{i=1}^{M}x_{i}$ and $2{\hat{\sigma }}^{2}=\frac{2}{M}\sum_{i=1}^{M}{{(x}_{i}-\hat{µ})}^{2}$ are unbiased estimates of the sample total. This equation reveals that the computational unit with lower energy function output has higher priority. Therefore, the computation of attention weights for each computational unit can be reduced to the inverse of $e_{t}^{min}$, i.e., $a_{t}$:

(4) $$a_{t}=\frac{1}{e_{t}^{min}}+0.5.$$

The use of the sigmoid activation function in SimAM may lead to slow learning or even stagnation due to too large or small input signals during the forward propagation. Therefore, in this paper, we use the scaling function Hard sigmoid for feature refinement, which is formulated as follows:

(5) $$Hard\;Sigmoid\left(x\right)=\left\{\begin{array}{c} \begin{array}{cc} 0 & x<-3 \end{array}\\ \begin{array}{cc} 0.2*x+0.5 & -3\leq x\leq 3 \end{array}\\ \begin{array}{cc} 1 & x>3 \end{array} \end{array}\right.,$$

where x is the input signal and Hard sigmoid is an approximate expression for the sigmoid activation function, which has the advantage that no power calculation is required and the input signal is gradient stable in the open interval from −3 to 3.

Finally, the whole process can be expressed as follows:

(6) $$\tilde{X}=Hard\;Sigmoid\left(A\right)⊙X,$$

where A groups all attentions $a_{t}$ across channels and spatial dimensions, and $X\in R^{C\times H\times W}$ is the input feature.

#### 2.2.2. SoftPool Bi-Level Routing Vision Transformer (SP-BiFormer)

In the real environment, apple leaf disease targets have low resolution, limited pixel area, and are characterized by tiny targets. In addition, some of these diseases are sparsely distributed on the leaves or have a tendency to cluster. In the prediction phase of the YOLOv7 model, the anchor frames generated by the prediction rely on NMS (non-maximum suppression) to filter out a large number of low-confidence borders, which tends to miss the tiny targets. Transformer self-attention is a type of attention mechanism. The design idea is to reduce the dependence on external information and to use the original feature information to encode the association information at different locations as much as possible to achieve attention focusing.

To this end, we propose the SP-BiFormer Block to optimize the ability of YOLOv7-tiny to focus on tiny targets, as in Figure 3c. It adds the information about tiny targets by associating the perceptual features of the tiny target and the context, and uses the broader contextual information in the scene to assist in inferring the location or class of the tiny target. Specifically, the SP-BiFormer Block is inspired by Sense Time Research’s proposal of a dynamic and query-aware sparse self-attentive module BiFormer [34]. The core of this self-attentive module is BRA (bi-level routing attention), which consists of region partition and input projection, region-to-region routing with directed graph and token-to-token attention. The key idea is to filter out unimportant key-value pairs to achieve fine-grained and sparse attention.

According to reference [33], we give a preliminary mathematical definition of a vision transformer. For a given 2D feature map $X\in R^{H\times W\times C}$, its attention mechanism can be constructed from the linear projection queries $Q\in R^{N_{q}\times C}$, keys $K\in R^{N_{m}\times C}$ and values $V\in R^{N_{m}\times C}$ of X:

(7) $$Attention\left(Q,K,V\right)=softmax\left(\frac{QK^{T}}{\sqrt{N}}\right)V,$$

where H (height), W (width) and C (channel) denote the height, width and number of channels of the input image, respectively. Q,K  and V are numeric vectors. Softmax function maps the input to the (0, 1) space. $\sqrt{N}$ is a scalar.

Next, we will introduce the three parts of BRA; the first is the “Region partition and input projection” part. The input feature map $X\in R^{H\times W\times C}$ is initially partitioned into S×S disjoint regions (assume that X is a square, that is, H equals W), and then the query, key and value vectors are obtained by the linear projection of X being partitioned:

(8) $$Q=X^{r}W^{q},K=X^{r}W^{k},V=X^{r}W^{v},$$

where $Q,K,V,\mathrm{and}\;X^{r}\in R^{S^{2}\times \frac{HW}{S^{2}}\times C}$; $W^{q},W^{k},\mathrm{and}\;W^{v}\in R^{C\times C}$ are the weight of each linear projection.

The second part of BRA, “Region-to-region routing with directed graph”, is presented next. This part calculates the regions that should be focused on by constructing a weighted directed graph from the input feature map X’s delineated regions. First, the average values of Q and K in each partitioned region are calculated separately to obtain $Q^{r}\;\mathrm{and}\;K^{r}\in R^{S^{2}\times C}$. Then, the adjacency matrix $A^{r}$ for the semantic correlation between regions is calculated:

(9) $$A^{r}=Q^{r}{\left(K^{r}\right)}^{T}.$$

To reduce the interaction times of each region with other regions, BRA keeps the k most relevant query regions for each region by index matrix $I^{r}\in N^{S^{2}\times k}$.

(10) $$I^{r}=topkIndex\left(A^{r}\right).$$

Compared with the conventional transformer, this operation can effectively reduce the amount of computation.

In the third part of BRA, “Token-to-token attention”, the key and value vectors are integrated for GPU (graphics processing unit) operations [33]:

(11) $$K^{g}=g\left(K,I^{r}\right),V^{g}=g\left(V,I^{r}\right),$$

where $g\left(\cdot \right)$ is the operation to gather the tensor.

Therefore, we can represent BRA according to the transformer self-attentiveness defined by Equation (7):

(12) $$BRA=Attention\left(Q,K^{g},V^{g}\right)+LE\left(V\right),$$

where LE(·) is a local enhancement operation of MTA (multi-scale token aggregation) on V by deep convolutional networks [43].

In order to provide sufficient information about small objects, we constructed the SP-Biformer Block based on BRA, which can utilize higher-level abstract features as the context of small targets and query contextual association information from the surrounding pixels of small objects. In BRA, average pooling for region-to-region routing is used to find the most relevant token regions. In SP-Biformer Block, we use SoftPool pooling instead of average pooling in the original BRA to reduce the redundancy of features. The SoftPool operation can retain. The output feature map of SoftPool is calculated by weighing the sum of all signal values in the candidate region R with the following equation:

(13) $$\tilde{a}=\sum_{i\in R}w_{i}*a_{i},$$

where R is the neighborhood region, $w_{i}=\frac{e^{a_{i}}}{\sum_{j\in R}e^{a_{j}}}$. Then, we sequentially embed the BRA module and the MLP (multi-layer perceptron) module to build the BiFormer Block for sparse relationship modeling of tiny targets.

#### 2.2.3. SIOU Loss

In supervised learning, the loss function as a penalty is the key to guide the model parameters to update correctly. The accuracy of prediction localization is measured in the YOLO algorithm by calculating the IOU (intersection and concurrency ratio) loss between the prediction frame and the ground truth. Existing methods rarely consider the direction of mismatch between the ground truth and the prediction box. This drawback often leads to slow convergence and reduced efficiency of the model. Tiny apple disease targets are difficult to be quickly calibrated in the final prediction process, and the presence of more perceptual regions leads to a larger number of redundant feature frames. In this paper, we use SIOU as a loss function to accelerate the tiny target matching of apple leaf diseases.

In particular, SIOU is defined as follows:

(14) $$L=1-IOU+\frac{C_{dis}+C_{shape}}{2},$$

where IOU denotes the ratio of the intersection of the prediction box and the ground truth box to the union set, and $C_{dis}$ and $C_{shape}$ are the distance cost and the shape cost that take into account the angle cost, respectively.

The distance cost is defined as follows:

(15) $$C_{dis}=\sum_{t=x,y}\left(1-e^{-\gamma \rho_{t}}\right)=2-e^{-\gamma \rho_{x}}-e^{-\gamma \rho_{y}},$$

where $\rho_{x}={\left(\frac{b_{cx}^{gt}-b_{cx}}{c_{w}}\right)}^{2}$, $\rho_{x}={\left(\frac{b_{cy}^{gt}-b_{cy}}{c_{h}}\right)}^{2}$, $\left(b_{cx},b_{cy}\right)$ and $\left(b_{cx}^{gt},b_{cy}^{gt}\right)$ are the center point coordinates of the prediction box and ground truth box, respectively; and $c_{w}$ and $c_{h}$ are the width and height of the minimum outer rectangle of the prediction box and ground truth box, respectively. $\gamma =2-C_{angle}$, where $C_{angle}$ is the angle cost, which defined as follows:

(16) $$C_{angle}=\cos\left(2\left(\sin\left(\alpha \right)-\frac{\pi }{4}\right)\right),$$

where α is the angle between the line connecting the ground truth box and the center of the prediction box and the horizontal or vertical line. In the training process, α is minimized if $\alpha <\frac{\pi }{4}$; otherwise, $\frac{\pi }{2}-\alpha$ is minimized.

The shape cost is defined as follows:

(17) $$C_{shape}={\sum_{t=w,h}\left(1-e^{-\omega_{t}}\right)}^{\theta },$$

where $\omega_{w}=\frac{\left|w-w^{gt}\right|}{max(w,w^{gt})}$, $\omega_{h}=\frac{\left|h-h^{gt}\right|}{max(h,h^{gt})}$; $\left(w,h\right)$ and $\left(w^{gt},h^{gt}\right)$ are the width and height of the prediction box and the ground truth box, respectively. θ is a constant, usually taken as 2 to 6 [38].

### 2.3. Evaluation Indicators

Herein, we use the classic *P* (precision), *R* (recall), mAP, AR, FPS, and parameter size to evaluate the performance of the model.

We first define the basic metrics *P* (precision) and *R* (recall). *P* represents the probability that the predicted category is correct; *R* represents the proportion of the number of samples correctly predicted to be positive to the total number that actually turned out to be positive. The relevant formulas are defined as follows:

(18) $$P=\frac{TP}{TP+FP}\times 100\%,$$

(19) $$R=\frac{TP}{TP+FN}\times 100\%.$$

where TP (true positive) represents positive samples predicted by the model to be in the positive category; FP (false positive) represents negative samples predicted by the model to be in the positive category; FN (false negative) represents positive samples predicted by the model to be in the negative category.

The *P*–*R* curve is formed by *P* and *R*, and its area is AP (average precision), reflecting the accuracy of a single class. mAP is an average of AP values for multiple prediction categories and is commonly used to measure the performance of models containing multiple categories in object detection. In this paper, the model is tested using an mAP value with an IOU threshold of 0.5. The calculation formula is as follows:

(20) $$mAP=\int_{0}^{1}P\left(r\right)dr.$$

The AR (average recall) is mainly used to measure the degree of model inspection failure. AR is calculated as follows:

(21) $$AR=\frac{R}{n}.$$

where *n* is the number of image frames detected.

FPS (frames per second) is an important measurement of detection speed. In this paper, it represents the average number of images detected per second. The formula for calculating FPS is as follows:

(22) $$FPS=\frac{1}{t}.$$

where *t* is the time required to process each image from input to output.

In addition, we use Params and GFLOPs to measure model size and computational complexity. Where FLOPs stands for floating point of operations, and one GFLOP is equal to one billion (=1 × 10^−9^) floating point operations per second.

## 3. Experiment Results and Analysis

### 3.1. Experimental Environment and Parameter Settings

The experiments in this paper are performed in the same hardware and software environment to ensure that the results are only relevant to the detector itself, as shown in Table 2. In order to increase the diversity of samples, we adopted Mosaic and Mixup data enhancement methods to improve the robustness of the network. The Mosaic method, derived from YOLOv4 [44], is a data enhancement method that produces a new image by randomly cropping four images and re-stitching them onto a single image. This can enrich the background of the image and reduce the dependence of batch size; Mixup, on the other hand, mixes two images proportionally into a new sample by linear interpolation. It can effectively prevent overfitting and improve the generalization ability of the model.

In the training process, we used the YOLOv7 pre-training model to initialize the parameters, speed up the initial training of the model and reduce the convergence period; FP16 mixed precision training was used to improve the throughput of single-sample training; this reduces the memory per iteration round and expands the number of batch sizes. Overfitting was suppressed by using the label smoothing regularization strategy, which can narrow the gap between hard and easy samples and improve model overfitting.

We divided the TTALDD-4 dataset by 8:1:1 into training set (5185), validation set (648), and test set (648) using common ten-fold cross-validation [45]. And the proportion of division was carried out in the same category. Herein, the training set was used to update the model parameters; the validation set was used to measure the optimization of the model but did not participate in the training; and the test set was only used to evaluate the model performance and also does not participate in the training. During the divisions, we unfolded across disease categories by randomizing the number of seeds. This was able to keep the high number of categories from interfering with the feature representation of the low number of categories, as the training, validation, and test sets were in the same proportion for each disease category. It is worth mentioning that after randomly dividing the dataset, the training, validation and test set image IDs for each detector were fixed, ensuring that the experimental results are only related to the performance of the detectors.

In addition, in real-world environments, the incidence of a fewer number of categories is lower and sample collection is difficult. If extensive digital enhancement is performed directly, the resulting image is very similar to the original image in terms of disease characteristics. For example, the affine transformation leaves the disease values essentially unchanged, with only a displacement of the disease region. This causes the network to be trained multiple times with the same disease features, falling into local optimization and producing overfitting. In order to ensure the generalization performance of the detector over these classes, we did not carry out further data enhancement strategies.

**Figure 4:**
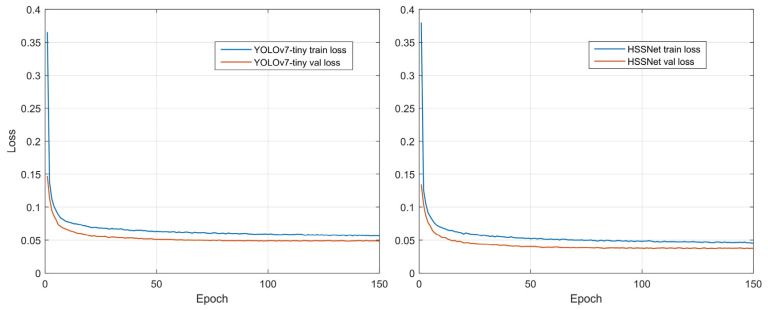
Training curve of YOLOv7-tiny and HSSNet.

Considering the hardware resource limitation, we set the Batch size to 32 (at this moment, the video memory was already full) and Epochs to 150 (the detector has converged at that number of rounds as in Figure 4); optimizations training was carried out by the SGD optimizer and cosine annealing strategy [46]. The learning rate first decreases slowly, then accelerates and finally decelerates again. This can help the network find the convergence interval quickly and speed up the convergence. Hence, the learning rate ranges from 1 × 10^−3^ to 1 × 10^−5^ and the decay rate is 5 × 10^−4^. We followed PyTorch’s default settings [47] and set Momentum to 0.9. The experimental parameters are shown in Table 3. In model testing, we use only test set images and the 150th epoch of convergence points of the detection model to ensure a fair comparison of test results.

### 3.2. Performance Comparison of HSSNet and YOLOv7-Tiny

To verify the optimization performance of HSSNet, we conducted comparative tests on the main performance indicators, as shown in Table 4. The results show that HSSNet is significantly higher than YOLOv7-tiny in both mAP and AR. The prediction box fits the disease area better and effectively classifies four different diseases. This is because H-SimAM helps YOLOv7-tiny adjust the weight of attention to annotated objects and similar backgrounds. In addition, SP-BiFormer improves the perception of tiny targets. Although the FPS of HSSNet decreased slightly compared to the baseline network, the value of 83 still ensured the high real-time performance of the application process. In addition, SP-BiFormer inevitably added Params due to the introduction of the transformer architecture, but based on the lightweight architecture of YOLOv7-tiny, the final model size of HSSNet is only 40 MB.

To verify the optimization performance of HSSNet, we conducted comparative tests on the main performance indicators, as shown in Table 4. On single-category APs, HSSNet improved the detection of a small number of categories (Alternaria leaf spot and Rust) while maintaining the detection accuracy of a large number of categories (Frogeye leaf spot and Grey spot). The results show that HSSNet is significantly higher than YOLOv7-tiny in both mAP and AR. The prediction box fits the disease area better and effectively classifies four different diseases. This is because H-SimAM helps YOLOv7-tiny adjust the weight of attention to annotated objects and similar backgrounds. In addition, SP-BiFormer improves the perception of tiny targets. Although the FPS of HSSNet decreased slightly compared to the baseline network, the value of 83 still ensured the high real-time performance of the application process. Besides, SP-BiFormer inevitably increases Params and GFLOPs due to the introduction of the Transformer structure. Among them, the increase in GFLOPs is small and has a weak impact on the model training time.

Figure 4 shows the training optimization process for both detectors. As can be seen from the curve changes, both YOLOv7-tiny and HSSNet converge at 150 epochs. Although HSSNet converges slightly slower than YOLOv7-tiny (converging to the convergence point at 50 epochs), its convergence point is lower. This indicates that HSSNet better expresses the disease features, has a better model fit, and is more suitable for tiny-target detection of apple leaf diseases.

In addition, the introduction of statistical tests can adequately assess whether HSSNet has a significant improvement in performance. For a disease image in the test set, we set the predicted label encodings (encodings consisted of IOU, localization and category values) and the true label encodings as two independent variables, and then performed a differentiation analysis on these two variables. Considering that disease location and category do not present a certain regular distribution on the images, we use the Wilcoxon Signed-Rank method [48] to carry out statistical tests. This method does not require the dependent variable to conform to a normal distribution.

In the Wilcoxon Signed-Rank hypothesis test results, the P value represents whether the error between the detector prediction and the manual labeling of the frames shows significance. If it presents significance (*p* < 0.05), it indicates that the gap between the model prediction results and the accurate labeled boxes is large, and the model prediction effect is poor; Cohen’s d value can measure the volatility of the detection, and the lower the value, the more stable the detection effect is. From the table, it can be seen that HSSNet is not only smaller than YOLOv7-tiny in detection error, but also the detection results are more stable, with better robustness and stability.

In summary, HSSNet loses some FPS and has an increase in Params and GFLOPs, but its excellent accuracy gains on a small number of categories are well worth it. In addition, based on the lightweight architecture of YOLOv7-tiny, the final model size of HSSNet is only 40 MB.

### 3.3. Module Validation Experiments

#### 3.3.1. H-SimAM

To improve the ability of the model to extract foreground object features in a complex background, we embed H-SimAM modules in different levels of the YOLOv7-tiny backbone network. The comparison results of attention modules in Table 5 show that the accuracy of the H-SimAM attention module is comparable to that of CBAM, but its zero-parameter overhead brings great convenience to the actual deployment of the model, and the module does not affect the detection speed. In SimAM, the introduction of the Sigmoid function may cause the problem of gradient disappearance, that is, degradation of the network. Therefore, we optimized with Hard Sigmoid. The results show that the introduction of Hard Sigmoid improves this problem and improves the detection accuracy.

#### 3.3.2. SP-BiFormer

To enhance the ability of YOLOv7-tiny to focus on tiny disease targets, we added an SP-BiFormer to the network prediction layer, which is located at layers P3, P4 and P5 of the feature pyramid, respectively. Their output feature map sizes are 80 × 80, 40 × 40 and 20 × 20, respectively. In SP-BiFormer, the input feature map is divided into multiple token query windows, and the size of the window will affect the parallel value search of the sparse effect. Therefore, we first tested the adaptability of different size windows (5 × 5, 10 × 10, 20 × 20) to tiny disease targets.

Table 6 shows that 10 × 10 size windows have the best overall effect. This is due to the fact that its query area is more adaptable to the range of valid values of small target features. The 5 × 5 size has more windows and more intensive queries. Correspondingly, parallel computation time also increases, making model detection slower. The 20 × 20 window is less numerous and more sparsely distributed, but the accuracy is slightly reduced. This is because although it is easier to detect the location of small targets, it coincides with the feature map region of layer P5, and does not highlight SP-BiFormer’s sparse perceptual query ability.

#### 3.3.3. SIOU

To optimize bounding box regression of tiny disease targets in training, we use the SIOU loss function. Table 7 shows the performance of different IOU losses on apple leaf diseases. It can be observed that the model’s mAP and AR are improved after SIOU is used, which indicates that it can better locate leaf lesions. In addition to this, we also tested the performance of GIOU and CIOU. The results show that both of them are inferior to SIOU in performance because SIOU pays attention to the characteristics of the angle and direction of the unmatched prediction frames.

### 3.4. Ablation Experiments

In order to verify the necessity of various improved algorithms for YOLOv7-tiny, we conducted ablation experiments on HSSNet, as shown in Table 8.

- As shown in Table 8, compared with group A, B, E and F, H-SimAM filters the complex background while maintaining FPS, effectively promoting supervised learning to pay attention to foreground disease objects, and there is no parameter quantity overhead.
- Comparing the A, C, E, and G experiments, it was found that SP-BiFormer decreased in FPS and improved the number of parameters and GFLOPs. However, its improvement in recall and precision is very significant, effectively distinguishing different categories of disease features and improving detection precision. Such a trade-off is very meaningful and worthwhile for small-target detection. Therefore, the embedding of this module is necessary for the detection of sparse tiny target diseases.
- Comparing group A, D, F, and G, SIOU optimizes the detection training process and improves the detection precision of the model. In addition, the inclusion of the angle loss metric is beneficial for the inference speed of the model.

In summary, H-SimAM inhibits unsupervised signals and improves the model’s effective focus on disease targets. SP-BiFormer is based on sparse perceptual query ability, which greatly promotes the ability of the model to detect tiny targets. SIOU considers the direction of the mismatch between the predicted box and the real box, and weighs the distance loss and angle loss in the design, which improves the model accuracy and improves the model’s training efficiency.

### 3.5. Visualization Results

We compared the performance effects of the baseline network YOLOv7-tiny and HSSNet in the typical scenarios under four categories, and the results are shown in Table 9. The detection category and confidence score are displayed in the prediction box.

In Table 9 (a), in the detection scene of the Alternaria leaf spots, the disease and the soil in the background were similar in color. Since YOLOv7-tiny did not add an attention mechanism to filter the confusing information, the missed detection occurred. HSSNet, with the help of H-SimAM, monitored the disease segment and showed good confidence.

In Table 9 (b), in the detection scenario of the Frogeye leaf spots, YOLO v7-tiny missed detection under light changes, while HSSNet detected objects under both strong and low light, with better robustness.

In Table 9 (c), in the detection scenario of the Grey spots, thanks to the sparsity of SP-BiFormer, the detection precision of HSSNet on apple leaf disease tiny targets is significantly improved compared with that of YOLOv7-tiny, and the network’s perception of apple leaf disease features is more stable.

In Table 9 (d), in Rust’s detection scenario, YOLOv7-tiny missed two lesions similar to the leaf background. HSSNet predicted both diseases with 53% and 61% confidence.

### 3.6. Comparison with Other Models

In addition, we also compare the performance of different models to explore the adaptability of HSSNet in tiny target apples. Table 10 shows that PPYOLOE-S is slightly better than YOLOv7-tiny in precision, but its FPS and Params are not dominant. YOLOv7-tiny has better balance in detection speed and accuracy, which is convenient for our practical application. This is also the reason why we chose it as a baseline. Compared with these networks, HSSNet has a good adaptability to tiny target diseases in apple leaves, and greatly improves the missing and false detection of tiny target diseases in YOLOv7-tiny.

In order to explore the scalability of the larger scale YOLOv7 detector on the dataset of this paper, we conducted related experiments using both the standard and X versions. The results show that the detection speed, precision, and recall decrease drastically as the model size increases. This suggests that the YOLOv7 version with a larger number of parameters suffers from severe overfitting and the model falls into some simple disease targets. Therefore, the YOLOv7-tiny model is a better fit for the application scenario of this task.

In addition, we compared other series of detectors of the same era as YOLOv7. In order to avoid the overfitting of detectors, we chose detectors that are similar to the tiny version in terms of model size and number of parameters. The experimental results show that RTMDet-S improves the backbone and neck network of the YOLO series and performs better in detection accuracy. However, it shows a severe decrease in FPS and an increase in the number of parameters and GFLOPs. Compared to the YOLOv7-tiny benchmark, it does not have an advantage in terms of overall performance. We also compare the lightweight detectors GhostNetV2 and MobileViT-S. Among others, GhostNetV2 has a great advantage in speed and the extreme lightness of the model makes the GFLOPs very small. However, this also loses the feature expression ability of the model, and thus the detection accuracy is poor. MobileViT-S, based on the transformer architecture, has better long-range modeling capability and is close to YOLOv7-tiny in small-target disease detection. However, the model loses serious inference speed as the current hardware does not have a dedicated transformer accelerator.

## 4. Discussion

The YOLOv7-tiny lightweight baseline network can rapidly detect apple leaf diseases with a certain accuracy. Based on this, we propose HSSNet to improve the detection precision of YOLOv7-tiny for apple leaf diseases with complex background features and tiny target features in natural environments, while ensuring high real-time detection speed.

**Figure 5:**
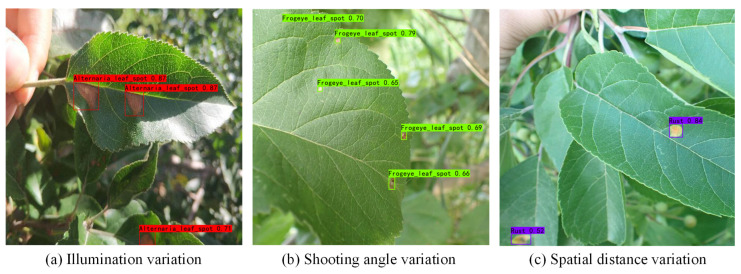
Advantages and disadvantages of HSSNet.

We selected some typical scenarios in the detection process to discuss the comprehensive performance of the model, as shown in Figure 5:

(a) When the lighting conditions changed, HSSNet still maintained robustness and detected tiny targets with high confidence;

(b) HSSNet missed or judged the location and type of disease with low precision when there was a certain angle between the front surface of the leaf disease and the front surface of the camera;

(c) HSSNet had difficulty in distinguishing the leaf disease with a deep distance when the foci of the leaf disease varied in spatial distance from the camera.

Although HSSNet achieved a high detection precision in the four types of apple leaf disease detection mentioned in this paper, it still has some limitations. In our detection example, there is a lack of datasets with multi-directional shooting views involved in the training and testing process, and the model robustness needs to be further optimized. In addition, the robustness of HSSNet in detection scenarios with multiple apple leaves and overlapping occlusions still needs to be verified. In view of the limited hardware resource budget in practical application environments, a more lightweight real-time detection network needs to be investigated.

## 5. Conclusions

Herein, to explore the optimization of apple leaf disease detection using deep learning object detection algorithms, we propose an improved HSSNet based on the baseline YOLOv7-tiny model. First, we constructed a precisely labeled image dataset, TTALDD-4, with four categories of apple leaf diseases by ourselves. For the complex background of apple leaf diseases, we propose that the H-SimAM zero-parameter attention module optimizes the ability of YOLOv7-tiny to focus on foreground targets. The structure can suppress the expression of unsupervised signals and prompt the network to focus on disease information more efficiently. For the characteristics of apple leaf disease mini-targets, we propose the BRA module-based SP-BiFormer Block to optimize the ability of YOLOv7-tiny to capture tiny-target disease information. This module aids in inferring small targets through associating context-aware features of disease small targets and using a wider range of features in the scene. Furthermore, with these implementations, we address the problem that it is difficult to quickly calibrate the true location of apple leaf disease tiny targets resulting in reduced training efficiency and detection accuracy. We use the SIOU loss function to optimize the model training. SIOU better fits the dense distribution features of apple leaf diseases in the training process and accelerates the inference process. The experimental results show that HSSNet achieves 85.04 (+4.67) in mAP and 67.53 (+7.68) in AR, and effectively reduces the cases of YOLOv7-tiny false detection and missed detection of small-target diseases. It must be noted that we have not incorporated variety-specific a priori knowledge into the optimization of the detector because different apple varieties were not taken into account. Nevertheless, HSSNet was able to perform well in the detection of small targets in apple leaves of mixed varieties.

In summary, HSSNet significantly improved the precision of the YOLOv7-tiny model in the field of apple leaf disease detection, while maintaining a balance between model parameters and detection speed. This research can assist apple growers to accurately apply pesticides based on the detection results. This provides a fresh reference for deep learning in ensuring modern pest control planting in the apple growing industry with practical applications.

## Figures and Tables

**Table 1:** The number and characteristics of apple leaf images.

Category	Example	Characteristics	Image Num	Label Num
Healthy	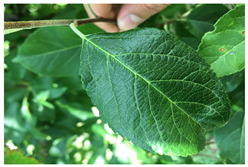	The color and texture of the leaves are soft green or dark green, with serrated leaf edges and clear vein textures.	\	\
Alternaria leaf spot disease	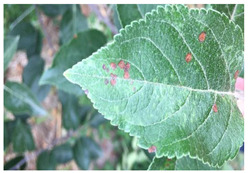	The disease shows dark brown or black leaf spots on the leaves that have a tendency to aggregate.	403/269	1728/1134
Frogeye leaf spot disease	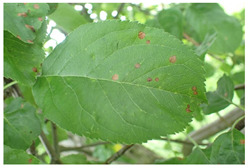	The lesions of the disease are brownish in color and are often distributed in spots covering the entire leaf.	3175/3175	15,735/11,806
Grey spot disease	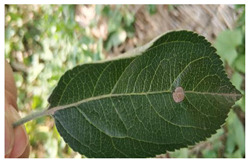	The disease initially appears as a sub-circular reddish-brown spot and later turns gray with small black spots scattered in the center.	337/161	742/312
Rust disease	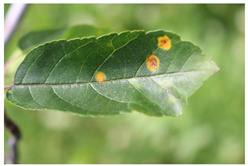	The disease lesions form shiny little spots with an orange-red color.	2566/2393	13,709/9254

**Table 2:** Hardware and software parameters.

**Hardware environment**	CPU	AMD EPYC 7642 48-Core Processor
	GPU	RTX 3090
	RAM	80 GB
	Video Memory	24 GB
**Software environment**	OS	Ubuntu 20.04
	CUDA Toolkit	V11.7
	CUDNN;	V8.2.1
	Python	3.8
	torch	1.11.0
	torchvision	0.12.0

**Table 3:** Training parameter settings.

**Input shape**	640 × 640	**Epochs**	150
**Batch size**	32	**Momentum**	0.9
**Learning rate**	1 × 10^−2^~1 × 10^−4^	**Weight decay**	5 × 10^−4^
**Lr decay type**	cosine	**Optimizer**	SGD

**Table 4:** Performance comparison between HSSNet and YOLOv7-tiny.

Metrics	YOLOv7-Tiny	HSSNet
AP (Alternaria leaf spot)	62.76	**75.69**
AP (Frogeye leaf spot)	93.16	**93.83**
AP (Grey spot)	77.21	**82.08**
AP (Rust)	88.36	**88.58**
mAP	80.37	**85.04**
AR	59.85	**67.53**
FPS	**97**	83
Params (M)	**6.227**	10.396
GFLOPs (G)	**13.4**	14.1
*p* value	0.950	**0.971**
Cohen’s d value	0.0557	**0.0482**

**Table 5:** Validation of H-SimAM.

Methods	mAP	AR	FPS	Params (M)	GFLOPs (G)
YOLOv7-tiny	80.37	59.85	97	6.227	**13.4**
+SE [27]	81.47	63.51	95	6.399	13.4
+CBAM [28]	82.82	65.48	96	6.399	13.4
+SimAM [29]	82.99	65.44	96	6.227	13.4
**+H-SimAM**	**83.36**	**66.02**	**97**	**6.227**	13.4

**Table 6:** Validation of SP-BiFormer.

Methods	mAP	AR	FPS	Params (M)	GFLOPs (G)
YOLOv7-tiny	80.37	59.85	**97**	**6.227**	**13.4**
+BiFormer (5 × 5) [34]	81.98	63.24	70	10.396	14.1
+BiFormer (10 × 10)	84.03	66.91	76	10.396	14.1
+BiFormer (20 × 20)	83.17	65.40	80	10.396	14.1
**+SP-BiFormer (10 × 10)**	**84.46**	**68.85**	78	10.396	14.1

**Table 7:** Validation of SIOU.

Methods	mAP	AR	FPS	Params (M)	GFLOPs (G)
YOLOv7-tiny (IOU)	80.37	59.85	**97**	**6.227**	**13.4**
+GIOU [36]	80.88	60.20	97	6.227	13.4
+CIOU [37]	81.55	62.47	98	6.227	13.4
+SIOU [38]	**82.72**	**64.85**	98	6.227	13.4

**Table 8:** Ablation experiments of HSSNet.

Groups	Methods	mAP	AR	FPS	Params (M)	GFLOPs (G)
A	YOLOv7-tiny (IOU)	80.37	59.85	97	6.227	13.4
B	+H-SimAM	83.36	66.02	97	6.227	13.4
C	+SP-BiFormer	84.46	68.85	78	10.396	14.1
D	+SIOU	82.72	64.85	98	6.227	13.4
E	+H-SimAM +SP-BiFormer	84.67	69.39	80	10.396	14.1
F	+H-SimAM +SIOU	83.65	67.25	94	6.227	13.4
G	+SP-BiFormer +SIOU	84.71	69.96	81	10.396	14.1
H	HSSNet	85.04	67.53	83	10.396	14.1

**Table 9:** Visualization comparison of HSSNet and YOLOv7-tiny.

Category	YOLOv7-Tiny	HSSNet
(a) Alternaria leaf spot	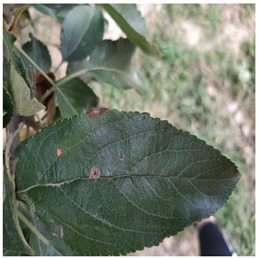	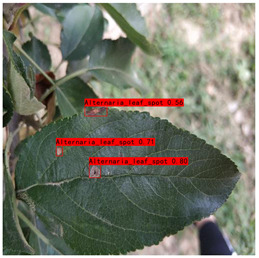
(b) Frogeye leaf spot	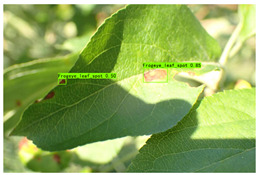	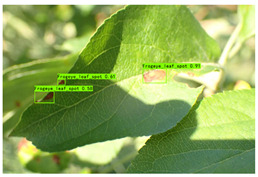
(c) Grey spot	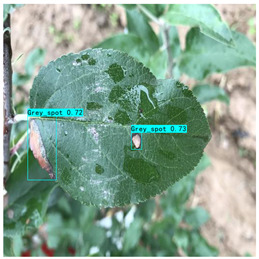	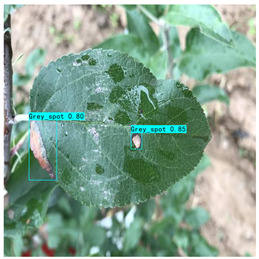
(d) Rust	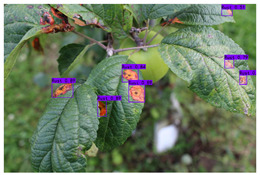	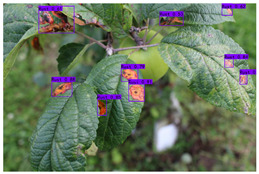

**Table 10:** Performance comparison of HSSNet with other detectors.

Detector	Backbone	mAP	AR	FPS	Params (M)	GFLOPs (G)
YOLOv5-S [49]	CSPDarkNet-53	78.34	57.67	81	7.269	16.5
YOLOX-S [50]	CSPDarkNet-53	80.87	60.29	72	8.968	26.8
PPYOLOE-S [51]	CSPRepResNet	81.15	61.30	90	7.959	17.4
YOLOv7-tiny	ELAN-CSPDarkNet	80.37	59.85	97	6.227	13.4
YOLOv7	ELAN-CSPDarkNet	75.59	53.05	59	36.912	104.7
YOLOv7-X	ELAN-CSPDarkNet	69.28	45.89	42	71.327	189.9
RTMDet-S [52]	Modified CSPDarkNet	82.83	61.73	68	8.990	14.8
GhostNetV2 [53]	GhostNetV2	77.39	55.18	122	6.154	0.2
MobileViT-S [54]	MobileViT-S	79.34	59.24	62	5.649	2.7
HSSNet	ELAN-CSPDarkNet	85.04	67.53	83	10.396	14.1

## Data Availability

Data available on request from the authors.
